# Combinatorial Cytokine Code Generates Anti-Viral State in Dendritic Cells

**DOI:** 10.3389/fimmu.2014.00073

**Published:** 2014-02-26

**Authors:** Boris M. Hartmann, Nada Marjanovic, German Nudelman, Thomas M. Moran, Stuart C. Sealfon

**Affiliations:** ^1^Department of Neurology, Mount Sinai School of Medicine, Center for Translational Systems Biology, New York, NY, USA; ^2^Department of Microbiology, Mount Sinai School of Medicine, Center for Translational Systems Biology, New York, NY, USA

**Keywords:** TNFa, IL1b, IFNb, anti-viral signaling, DC maturation, combinatorial effect

## Abstract

The physiological function of the immune system and the response to therapeutic immunomodulators may be sensitive to combinatorial cytokine micro-environments that shape the responses of specific immune cells. Previous work shows that paracrine cytokines released by virus-infected human dendritic cells (DC) can dictate the maturation state of naïve DCs. To understand the effects of paracrine signaling, we systematically studied the effects of combinations cytokines in this complex mixture in generating an anti-viral state. After naïve DCs were exposed to either IFNβ or to paracrine signaling released by DCs infected by Newcastle disease virus (NDV), microarray analysis revealed a large number of genes that were differently regulated by the DC-secreted paracrine signaling. In order to identify the cytokine mechanisms involved, we identified 20 cytokines secreted by NDV infected DCs for which the corresponding receptor gene is expressed in naïve DCs. By exposing cells to all combinations of 19 cytokines (leave-one-out studies), we identified five cytokines (IFNβ, TNFα, IL-1β, TNFSF15, and IL28) as candidates for regulating DC maturation markers. Subsequent experiments identified IFNβ, TNFα, and IL1β as the major contributors to this anti-viral state. This finding was supported by infection studies *in vitro*, by T-cell activation studies and by *in vivo* infection studies in mouse. Combination of cytokines can cause response states in DCs that differ from those achieved by the individual cytokines alone. These results suggest that the cytokine microenvironment may act via a combinatorial code to direct the response state of specific immune cells. Further elucidation of this code may provide insight into responses to infection and neoplasia as well as guide the development of combinatorial cytokine immunomodulation for infectious, autoimmune, and immunosurveillance-related diseases.

## Introduction

The limitations of single cytokine therapy have motivated interest in evaluating the effects of combinatorial treatment. Individual therapeutic cytokines often fail to achieve full or sustained clinical benefits for many patients. For example, IFNα, which is the current therapy for chronic hepatitis C infection, fails to clear HCV titers in half of treated patients (1). The cytokine interferon beta (IFNβ) has limited activity against multiple sclerosis in a large segment of patients (2). Cytokine combination therapy, where two or more cytokine-based medications are simultaneous administered to treat a single disease, has shown promise in multiple medical conditions, such as cancer (3), myocardial infarction (4), and osteoporosis (5). Recent studies have also begun to reveal how combined extracellular stimuli can synergistically direct the responses of immune cells. Retinoic acid combined with IL-15 causes dendritic cells (DCs) to skew the T-cell polarization toward TH17 cells (6). SCF and IL-2 have a synergistic effect on the proliferation NK cells (7). TNFα and IFNγ act together on smooth airway cells to enhance CXCL-10 expression (8). IL17 together with TNFα or IL1β induces MCP-1 and MIP-2 in murine mesangial cells (9). Despite its potential, studying combinations of cytokines is experimentally difficult and relatively little systematic exploration in this important area has been reported.

We previously reported that paracrine signaling mediated by the complex mixture of cytokines secreted by virus-infected DCs in culture causes naïve uninfected DCs to develop an anti-viral state characterized by upregulation of DC maturation markers, increased phagocytic activity, and greater resistance to viral infection (10). Since the discovery of type I interferon, paracrine cytokine signaling has been recognized as a crucial component in orchestrating the immune responses to virus infection. However, IFNβ pretreatment alone is not sufficient to induce this paracrine induction of anti-viral activated DCs (10). In the present study, we investigate the combinatorial cytokine code underlying this effect, by studying combinations of the single components of the secretome of virus-infected DCs. Understanding how this combinatorial cytokine code modulates immune responses may guide the development of better combination therapy approaches and help elucidate how the microenvironment directs appropriate responses in specific cell types during infection.

## Materials and Methods

### Differentiation of DCs

All human research protocols for this work have been reviewed and approved by the IRB of the Mount Sinai School of Medicine. Monocyte-derived DCs were obtained from healthy human blood donors following a standard protocol described elsewhere (11). Briefly, human peripheral blood mononuclear cells were isolated from buffy coats by Ficoll density gradient centrifugation (Histopaque, Sigma Aldrich) at 1450 rpm and CD14^+^ monocytes were immunomagnetically purified by using a MACS CD14 isolation kit (Miltenyi Biotech). Monocytes were then differentiated into naïve DCs by 5–6 days incubation at 37°C and 5% CO_2_ in DC growth media, which contains RPMI Medium 1640 (Invitrogen/Gibco) supplemented with 10% fetal calf serum (Hyclone), 2 mM of l-glutamine, 100 U/mL penicillin and 100 g/mL streptomycin (Pen/Strep) (Invitrogen), 500 U/mL hGM-CSF (Preprotech), and 1000 U/mL hIL-4 (Preprotech). All experiments were replicated using cells obtained from different donors. Overall, we used DCs from 21 different donors for this study.

### Virus preparation and viral infection

The Newcastle disease virus (NDV) (rNDV/B1) was generated in Prof. Peter Palese’s laboratory (12). NDV-RFP, Influenza A/California/04/09 (H1N1), and A/Puerto Rico/8/1934 (H1N1) were obtained from Prof. Adolfo Garcia-Sastre’s laboratory (13). For infection, virus stocks were diluted in serum free medium and added directly onto pelleted DCs at a multiplicity of infection of 1.

### Generation of AVDCs

Anti-viral activated dendritic cells (AVDCs) were generated by employing a trans-well system. The trans-well system consists of an upper and a lower chamber separated by a 0.4 μm PET membrane (Millipore) that allows diffusion of cytokines and chemokines through the membrane but avoids the interaction of the cells in both chambers. To generate the AVDCs, naïve DCs were infected as described above. After the 40 min incubation, the cells were washed with PBS, and cultured in the trans-well system. Infected and non-infected DCs were allocated in the upper and lower chamber, respectively. Two independent wells were set-up with infected or naïve non-infected DCs as positive and negative controls. The cultures were incubated at 37°C in 5% CO_2_ for 18 h. All cells were then washed in PBS and harvested for flow cytometry analysis and RNA isolation. The supernatant was kept at −80°C for ELISA analysis of cytokines/chemokines.

### Microarray analysis

Samples from AVDCs, DCs infected with NDV, and DCs treated with IFNβ for 8 h were used for microarray analysis. Naive DCs served as negative control. Three samples were taken per treatment. RNA was extracted with the RNeasy plus kit (Qiagen) following the manufacturer’s protocol. Gene expression was assayed using broad human genome specific HG-U133_Plus_2 GeneChip expression probe arrays (Affymetrix). Raw data was processed with the Partek Pro software using the RMA background correction, with an adjustment of GC content as pre-background adjustment. Data was normalized to its quantile, data was log transformed to a base of two, and probe sets were summarized to its mean. Principal component analysis (PCA) of samples plotted in genespace was performed for all probe sets. Robustness of the PCA was tested by randomization (Figure S1 in Supplementary Material). One-way ANOVA was calculated by using Method of Moments (14). Fisher’s least significant difference with FDR as multiple testing correction was used to calculate the following contrasts AVDC vs. IFNβ, AVDC vs. CTRL, IFNβ vs. CTRL, NDV vs. CTRL. List were generated by a fold change and *p*-value (FDR adjusted) criteria. Bioinformatic analysis was performed using Ingenuity software. The data used are deposited in NCBI’s gene expression omnibus (15) and are accessible through GEO series accession number GSE52081 (http://www.ncbi.nlm.nih.gov/geo/query/acc.cgi?acc=GSE52081).

### ELISA

In order to minimize the supernatant volume to assay, a Beadlyte Human Multiplex ELISA analysis (Millipore) was used following manufacturer instructions. Briefly, 100 μl from each compartment/well was incubated in a 96-well filter PVDF 1.2 μm plate specially designed to retain cytokines/chemokines, with a mixture of anti-cytokine IgG conjugated beads for the different cytokines/chemokines assayed. After 2 h incubation, the plate was filtered and washed three times with Assay solution (PBS pH 7.4 containing 1% BSA, 0.05% Tween-20, and 0.05% sodium azide). The washes were followed by 1.5 h incubation with biotin-conjugated anti-cytokine IgG. After Assay solution washing, Streptavidin–Phycoerythrin, was added followed by addition after 30 min Stop solution [0.2% (v/v) formaldehyde in PBS pH 7.4]. The plate was then filtered and each well resuspended in 125 μl of Assay buffer, and read in a Luminex 100 machine. Single cytokine ELISA (IFNβ) was also performed according to manufacturers protocol (PBL).

### Cytokine treatments

Dendritic cells were exposed to 1.3 μg/mL TNFα (Symansis), 9 μg/mL CCL3 (Symansis), 3.8 μg/mL IL8 (Symansis), 20 μg/mL CXCL10 (Peprotech), 0.5 μg/mL CCL5 (Peprotech), 9 μg/mL IL6 (Peprotech), 2.8 μg/mL IFNα2 (PBL InterferonSource), 0.03 μg/mL CXCL12 (Peprotech), 2 μg/mL IFNALPHA16 (PBL InterferonSource), 0.03 μg/mL IL12a (Symansis), 4.4 μg/mL IL18 (R&D SYSTEMS), 0.2 μg/mL IL1a (R&D SYSTEMS), 1 μg/mL IL1RA (R&D SYSTEMS), 4 μg/mL IL28a (AbD Serotec), 4 μg/mL IL29 (R&D SYSTEMS), 0.1 μg/mL TNFSF15 (AbD Serotec), 0.1 μg/mL TNFSF4 (R&D Systems), 0.1 μg/mL TNFSF10 (R&D Systems), 0.2 μg/mL IL1β (eBiosciences), and 8.89 μg/mL IFNβ (PBL InterferonSource) in various combinations for 8 h. For the first screening with 20 cytokines, we used cells from three different donors. To adjust for overall differences between individuals, we normalized data of each individual to the median of all treatments. All other cytokine screening experiments were carried out with replicated from the same donor and then repeated with at least two additional donors.

### Flow cytometry analysis

Cells were washed with FACS staining buffer (Beckman Coulter) and stained with monoclonal antibodies for HLA-DR and CD86 (BD Biosciences). NDV-GFP cells were analyzed without any additional staining. Cells were assayed on an LSRII flow cytometer (BD Biosciences) and analyzed with Cytobank software (16). Raw data as well as analyses can be downloaded at: https://www.cytobank.org/cytobank/experiments?project=565

### Imaging flow cytometry analysis of bead uptake and apoptosis

For analysis for apotosis and infectivity cells were fixed after treatment with 1% paraformaldehyde (Electron Microscopy Science), permeabilized with Methanol (Sigma), and washed in PBS and stained with influenza NP specific antibodies (Abcam) and Hoechst 33342 (Invitrogen) as nuclear dye. Single cell images were acquired using the IS 100 Imaging flow cytometer (Amnis). Apoptotic cells were identified by fragmentation of nucleus (intensity of nuclear image at a 30% threshold) and shape of the brightfield image (contrast) using IDEAS software (Amnis). To detect phagocytosis, 1 μm 488 nm fluorescence labeled latex microspheres (Polysciences Corp.) at a concentration of 50 beads per cell were co-cultured for 2 h at 37°C with cytokine pretreated cells. Single cell images were acquired using extended depth field imaging distortion in order to identify beads in different focal planes within a cell. The numbers of beads incorporated by cells were quantified in the images captured using image analysis software (IDEAS Software, Amnis Corp).

### Real-time PCR

mRNA expression levels were quantified by real-time reverse transcriptase polymerase chain reaction (PCR). RNA was isolated from cells using Qiagen Micro RNeasy kit following the manufactures protocol (QIAGEN). cDNA was synthesized from total RNA with AffinityScript™ Multi-Temp RT (Stratagene) with oligo dT_18_ as primer. For real-time PCR PlatinumTaq DNA polymerase (Invitrogen) and a SYBR green (Molecular Probes) containing buffer were used. The real-time PCRs were performed using a thermocycler (ABI7900HT; Applied Biosystems) as previously described (21). The RNA levels for the house keeping genes ribosomal protein S11, tubulin, and β-actin were also assayed in all samples to be used as an internal controls. mRNA measurements were normalized using a robust global normalization algorithm. All control crossing threshold (Ct) values were corrected by the median difference in all samples from Actb. All samples were then normalized by the difference from the median Ct of the three corrected control gene Ct levels in each sample, with the value converted to a nominal copy number per cell by assuming 2500 Actb mRNA molecules per cell and an amplification efficiency of 93% for all reactions. PCR results from DCs exposed to combinations of cytokines were normalized to values from untreated cells and log 2 transformed prior further statistical analysis. To get a picture of overall induction of those genes assayed, we summarized the log transformed expression levels on the most right column of Figure 7. Primers for genes can be found in the Table S3 in Supplementary Material.

### T-cell activation assay

PBMCs were exposed to inactivated native measles virus for 4 days. From these samples, CD3 cells were isolated by negative selection using the Pan T-Cell Isolation Kit II (Miltenyi) and stained with CFSE (Invitrogen). Those cells were then co-cultured with cytokine pretreated DCs which were also pulsed with inactivated native measles virus. T-cell proliferation was measured by the reduction of CFSE intensity of cells.

### *In vivo* experimentation

Animal studies were performed in compliance with the U.S. Department of Health and Human Services Guide for the Care and Use of Laboratory Animals and protocols were approved by the Institutional Animal Care and Use Committee (IACUC) of the Mount Sinai School of Medicine. Animals were pretreated with murine 3.5 mg/kg BW IFNβ (PBL InterferonSource), murine 1.3 mg/kg BW TNFα (Peprotech), and 0.5 mg/kg IL1β (Peprotech) 6 and 3 h prior infection with the influenza A strain PR8 in an inhalation chamber.

### Statistical analysis

Micro array analysis was performed with the Partek Pro software 1-way ANOVA was calculated by using Method of Moments (14). Genes were compared by asymptotic unpaired *t*-test comparisons followed by a Benjamini–Hochberg multiple testing correction. All other data was analyzed with R. Maturation marker expression, apoptosis induction, and infectivity levels were first analyzed with ANOVA, followed with pairwise comparisons using the Tukey’s “Honest Significant Difference” method. PCR of the gene expression after combinatorial treatment was also analyzed with ANOVA followed Tukey’s “Honest Significant Difference” method and an additional Bonferroni multiple testing correction for the summarized data. Bead uptake data were analyzed with a pairwise Wilcoxon Rank Sum Test with the Bonferroni method for multiple testing correction. Survival of mice was analyzed with a Mantel–Haenszel test for survival analysis. Data as well as the R analysis can de downloaded from the supplementary data.

## Results

### Exposure to paracrine signaling from infected DCs induces gene expression profile distinct from that caused by IFNβ alone

**Figure 1:**
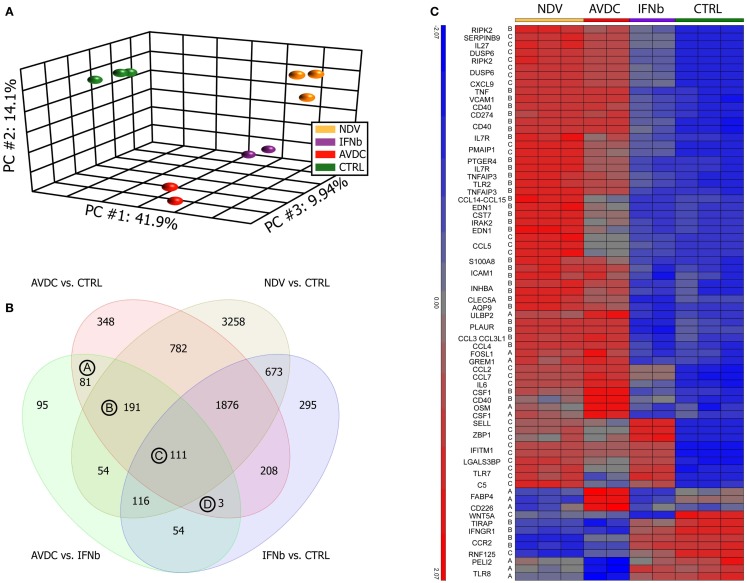
**Paracrine signaling from virus-infected DCs cells induce a different gene expression pattern compared to cells exposed to IFNβ alone**. Microarray profiles were obtained from human monocyte-derived DCs exposed to paracrine signaling from NDV infected DCs or to IFNβ alone. Sample groups: NDV: DCs infected with NDV; IFNβ: DCs exposed to IFNβ alone; AVDCs: DCs exposed to paracrine signaling from NDV infected cells in a trans-well system; CTRL: naive unexposed and uninfected DCs. **(A)** Principal component analysis of samples on all genes represented on the microarray. First three principal components showing 41.9% (PC1), 14.1% (PC2), and 9.94% (PC3) of the overall change in gene expression in the data set. **(B)** Venn diagram comparing genes which differed (twofold, FDR *p* < 0.05) between each pair of conditions. **(C)** Genes which are differed between AVDC vs. CTRL and AVDC vs. IFNβ and have a gene ontology association with anti-viral immune processes.

The effects of paracrine signaling from infected DCs on naive DCs, transforming them to what we have previously referred to as anti-viral activated DCs [AVDC, Ref. (6)], were studied using microarrays (Figure 1). To compare the effects of paracrine signaling to the effects of a single cytokine treatment, we exposed naive DCs either to paracrine signaling from NDV infected cells (which generates AVDCs) or to IFNβ at a concentration found in the supernatants of NDV infected DCs. RNA samples from naive cells (CTRL) and NDV infected cells (NDV) were also assayed.

A PCA on the samples was performed in order to test how much individual samples are similar to the biological replicates within a group and how the different groups relate to each other. The PCA showed that cells exposed to paracrine signaling from infected DCs had a different overall expression profile than cells exposed to IFNβ alone (Figure 1A). Top genes for vectors were CXCL11, ISG20, ISG20, IDO1, IFI27, IFITM1, IFIT2, OASL, and CXCL9 for principal component (PC) 1; IFIT2, CXCL11, CCDC88A, NEXN, MALAT1, TNFSF10, P2RY12, SAMD9L, SMCHD1, and NEXN for PC2 PPBP, MMP1, ADAM12, IRG1, AKAP12, SLC28A3, DNAJC6, FABP4, ITGA9, and FABP4 for PC3. ANOVA (FDR *p* < 0.05) and twofold change threshold relative to control cells identified 7088 genes altered by NDV infection, 3600 genes altered by paracrine signaling (AVDC), and 3336 genes changed by IFNβ alone.

The number of genes differentially expressed between each pair of the four groups studied is indicated in Figure 1B. A comparison between cells exposed to paracrine signaling and IFNβ alone showed 705 differentially expressed genes. From those 705 transcripts, which showed a significant change between exposure to paracrine signaling and single cytokine IFNβ treatment, 81 were significantly altered by the paracrine signaling but did not show significant induction by NDV infection or IFNβ treatment when compared to control [Group (A) in Figure 1B], 191 genes were significantly induced by paracrine signaling and NDV infection but not IFNβ treatment when compared to control [Group (B) in Figure 1B] and 111 genes were significantly induced by paracrine signaling, NDV infection, and IFNβ treatment when compared to control [Group (C) in Figure 1B]. Three genes were changed by paracrine signaling and IFNβ treatment when compared to control but still differed significantly when compared between exposure to paracrine signaling and IFNβ treatment. Heat maps of all genes in groups A, B, C, and D can be seen in the supplementary material (Figure S2 in Supplementary Material). Fifty genes from the list of 389 transcripts being significantly different when exposed to paracrine signaling vs. IFNβ alone, as well as when exposed to paracrine signaling and naive cells could be linked to anti-viral immunity (Figure 1C). Among those, 50 genes were regulators of inflammation and immune response including *VCAM-1* (17), *AQP9* (18), *RIPK2* (19), *IRAK2* (20), *CCL3L1* (21); cytokines like *IL6*, *OSM* (22); genes linked to anti-viral immunity *CCL3L3* (23), *CSF1* (24), *CD274* (25), *CD40* (26), *IL7R* (27); immune cell activation *CLEC5A* (28), *EDN* (29), *CST7* (30), and also a suppressor of apoptosis *PTGER4* (31).

### Bystander DCs are exposed to a complex cytokine environment

**Figure 2:**
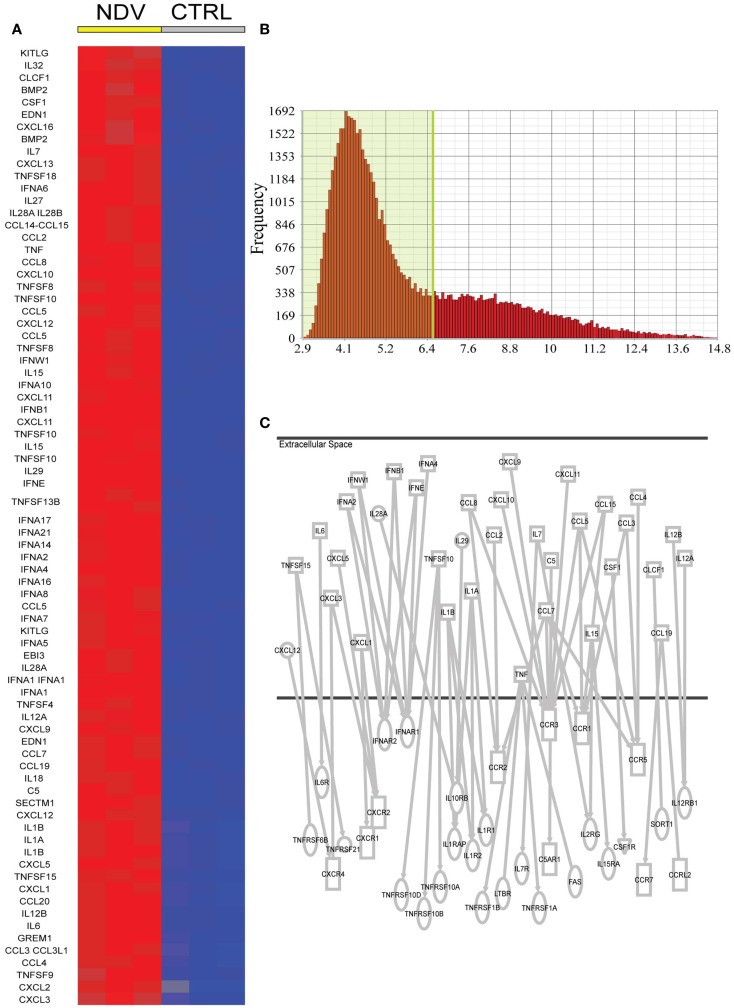
**Candidate NDV induced cytokines in DCs for a paracrine effect on DCs**. **(A)** Cytokines and chemokines induced by NDV (twofold FDR *p* < 0.05) compared to naive DCs. **(B)** Expression level of all genes expressed in naive DCs. A cutoff of expression above 6.5 was used to identify potentially expressed cytokine and chemokine receptors. **(C)** Ingenuity analysis linking induced cytokines/chemokines to receptor genes also expressed in DCs.

To identify cytokines and chemokines induced during NDV infection, to which uninfected bystander DC cells are exposed, we analyzed the 7088 transcripts induced by NDV. Seventy-eighty transcripts could be associated to the gene ontology terms cytokine activity or chemokine activity (Figure 2A). We further narrowed this list by setting an expression threshold of 6.5 based on the intersection of the two populations of expressed and non-expressed genes (Figure 2B) and identifying which cytokines/chemokines could be associated with receptor genes also expressed in DCs. This analysis linked CCL4 to CCR5 (32, 33), CCL3 to CCR5 (34), CCL3 to CCR1 (35), CCL2 to CCR2 (36, 37), CCL7 to CCR5 (33), CCL7 to CCR3 (38), CCL8 to CCR2 (39), CXCL10 to CCR3 (40), CXCL9 to CCR3 (40), CXCL11 to CCR3 (40), CCL15 to CCR3 (41), CCL15 to CCR1 (41), CCL5 to CCR3 (36), CCL5 to CCR5 (42, 43), CXCL12 to CXCR4 (44), CXCL3 to CXCR2 (36), CXCL5 to CXCR2 (45), CXCL1 to CXCR2 (46), IL6 to IL6R (47), IL1a to IL1R1 (48), IL1β to IL1R2 (49), IL1β to IL1R1 (50), TNF to TNFRSF1B (51), TNF to FAS (52), TNF to TNFRSF1A (53), IL15 to IL15RA (54), IL7 to IL7R (55), IL7 to IL2RG (56), IFNβ to IFNAR1 (57), IFNβ to IFNAR2 (58), IFNW1 to IFNAR1 (59), IFNA2 to IFNAR (60), IFNE to IFNAR1 (61), TNFSF10 to TNFRS10B (62), CCL19 to CCR7 (63), TNFSF15 to TNFRSF6B (64), IL28A to IL10RB (65), IL29 to IL10RB (65), IL12A to IL12RB1 (66), IL12A to IL12RB1 (67), and CSF1 to CSF1R (68) (Figure 2C).

**Figure 3:**
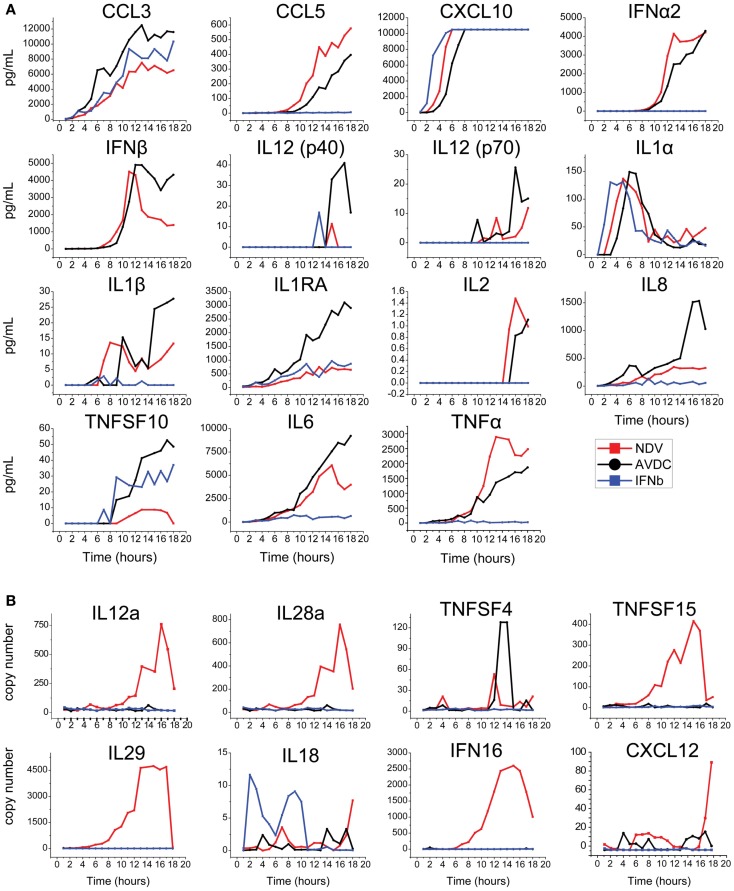
**Time course of expression of cytokines/chemokines in NDV infected DCs, AVDC, and IFNβ exposed DCs determined by (A) multiplex ELISA or IFNβ ELISA or (B) real-time PCR over an 18 h period**. NDV (red line) indicates measurements from supernatants (ELISA) or cells (PCR) which were infected with NDV at an MOI of 1. AVDC (black line) indicates measurements from supernatants from infected and co-cultured naïve DCs at a 1:1 ratio (ELISA) or naïve DCs which were co-cultured with infected cells (PCR). IFNβ (blue line) indicates measurements from supernatants (ELISA) or cells (PCR) from naïve DCs exposed to IFNβ alone.

The level of expression of cytokines and chemokines identified by this bioinformatics analysis was measured in an 18-h time course experiment (1) in supernatant from DCs infected by NDV, (2) in the supernatant associated with AVDCs in trans-well experiments, and (3) in supernatant of cells exposed to IFNβ alone by ELISA (Figure 3A) or in cellular mRNA by real-time PCR (Figure 3B). Cytokines which did not exhibit detectable expression by ELISA or PCR (not shown) were excluded for further screening. This led to the selection of the following 20 cytokines and chemokines for further study that were induced in NDV infected DCs: TNFα, CCL3, IL8, CXCL10, CCL5, IL6, IFNα CXCL12, IFNALPHA16, IL12a, IL18, IL1RA, IL28, IL29, TNFSF15, TNFSF4, TNFSF10, IL1α, IL1β, and IFNβ.

### Identification of individual cytokines contributing to combinatorial effects

We next studied the combinatorial effects of the 20 cytokines identified above on the induction of maturation marker expression in naïve DCs. Because studying all combinations of 20 cytokines was impractical, we identified combinatorial cytokine candidates by comparing the effect of all 20 cytokines on naïve DCs to the effects of all possible 19-cytokine combinations lacking one of the cytokines. These experiments used the maximum concentration measured by ELISA or, for cytokines measured by PCR, the concentration was estimated from transcript levels by comparing the PCR and ELISA levels of IFNβ. Many cytokines peaked at about 10 h during the 18 h time course. Therefore, we expose DCs to the cytokine mixtures for 8 h to best approximate the conditions of the paracrine signaling during viral infection. In this experiment, we used cells from three different donors for biological replicates, which resulted in a high variance of marker expression between donors. To adjust for differences between individuals, we normalized data to the overall median values.

**Figure 4:**
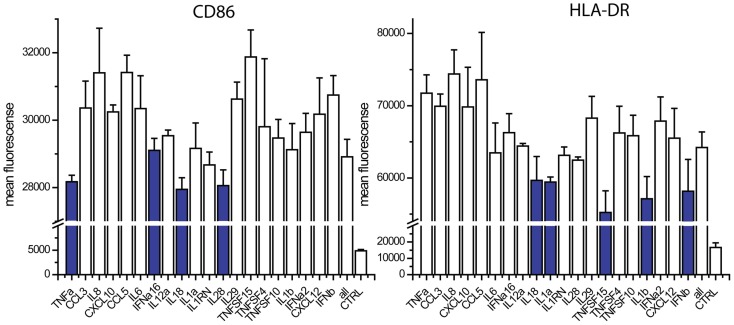
**Contribution of individual cytokines in 20 cytokine paracrine signal**. Maturation marker expression of DCs after 8 h to exposure to all 20 cytokines (all) or the leave-one-out combinations of 19 cytokines. Note that a reduction in expression with a cytokine absent indicates that the cytokine may contribute to induction.

**Figure 5:**
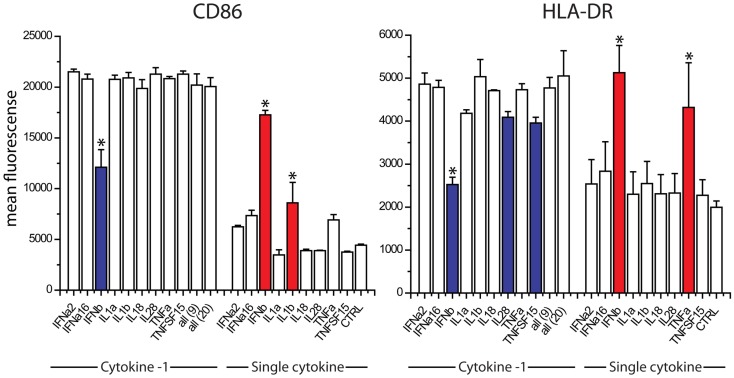
**Contribution of individual cytokines in nine cytokine paracrine signal**. Maturation marker expression of DCs after 8 h to exposure to all nine cytokines, all leave-one-out combination of eight cytokines and each individual cytokine treatment (Single cytokine) (*p ≤ 0.002 to cells exposed to all nine cytokines in Cytokine-1 treatment and to untreated cells in the single cytokine treatments).

The absence of TNFα, IL18, IL28, and IFNalpha16 reduced the expression of CD86, when compared to the exposure to all 20 cytokines (Figure 4). The absence of IFNa2, IL18, IL1α, TNFSF15, IL1β, and IFNβ reduced the expression of HLA-DR (Figure 4). We studied nine cytokines (IFNα, IFNALPHA16, IFNβ, IL1α, IL1β, IL18, IL28, TNFα, TNFSF15) in similar leave one cytokine out experiments as well as single cytokine exposure studies. These nine cytokines gave the same responses as the original 20, indicating that the 11 cytokines excluded from further study are not major contributors to maturation marker induction during paracrine signaling (Figure 5). The cytokine minus one studies with the remaining nine cytokines suggested the importance of IFNβ, for CD86 upregulation and IFNβ, IL28, and TNFSF15 for HLA-DR upregulation. When DCs were exposed to individual cytokines, IFNβ and IL1β induced CD86 and IFNβ and TNFα induced HLA-DR. Therefore five cytokines (IFNβ, IL28, TNFSF15, TNFα, IL1β) were selected for further study.

### TNFα, IFNβ, and IL1β induce a paracrine activated anti-viral state

**Figure 6:**
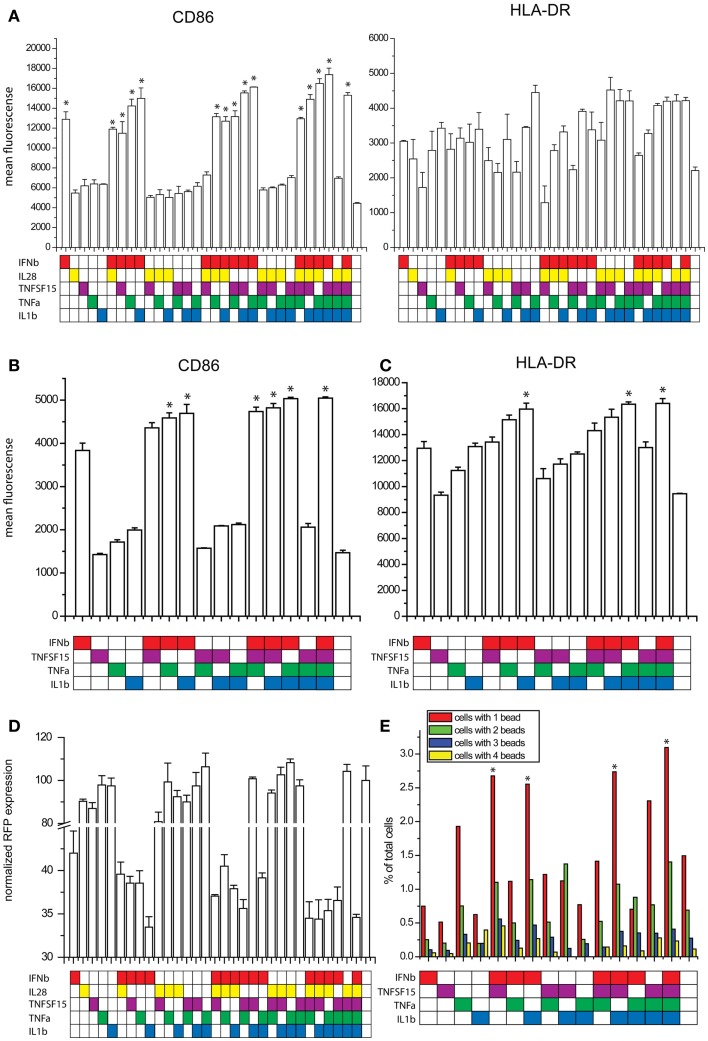
**Effects of combinatorial cytokine treatment**. **(A)** Maturation marker expression after exposure to combinations of IFNβ, IL28, TNFSF15, TNFα and IL1β, measured by flow cytometry (*p ≤ 0.05 compared to untreated cells). **(B)** CD86 expression after exposure to combinations of IFNβ, TNFSF15, TNFα and IL1β, measured by flow cytometry (*p ≤ 0.05 compared to cells treated with IFNβ alone). **(C)** HLA-DR expression after exposure to combinations of IFNβ, TNFSF15, TNFα and IL1β, measured by flow cytometry (*p ≤ 0.05 compared to cells treated with IFNβ alone). **(D)** Infectivity of NDV after cytokine combination pretreatment for 8 h in DCs measured by RFP expression. **(E)** Phagocytosis assay measured by bead uptake by imaging flow cytometry (*p ≤ 0.05 compared to untreated cells).

We studied the effects of combinations of the five cytokines on maturation marker expression, viral resistance, and phagocytic activity. For the maturation marker expression studies, human DCs were exposed to combinations of the five cytokines for 8 h and the levels of CD86 and HLA-DR were measured by flow cytometry. IFNb alone increases the expression of both markers. The additional increases observed with all combinations of cytokines did not achieve statistical significance in comparison to IFNb alone with tight control for family wise error. IL28 did not cause any trend toward an increase in maturation marker expression (Figure 6A). To improve statistical power, we performed maturation marker induction experiments using four cytokines IFNβ, TNFSF15, TNFα, and IL1b. Here, the combinations of IFNβ with either TNFα or IL1b showed a significantly higher induction of CD86 when compared to the effects of IFNβ alone (Figure 6B). The combination of IFNβ with IL1b showed a significantly higher HLA-DR induction when compared to IFNb alone (Figure 6C).

As one of the most important features observed in paracrine activated AVDCs is resistance to viral infection (6), we pretreated DCs with combinations of the five cytokines for 8 h and subsequently infected them with an RFP expressing NDV for 8 h. Infectivity was measured by using flow cytometry to quantify RFP expression. Because infectivity by RFP-NDV is so sensitive to IFNβ, its concentration was reduced for this study. Still, due to the effect of the RFP insertion which makes the virus less viable and more susceptible to the effects of IFNβ, changes observed with cytokine combinations were statistically not significant when compared to single cytokine IFNβ exposure. The combination of IFNβ and IL1β showed the largest reduction of infection (Figure 6D).

Another feature of AVDCs is the heightened phagocytic activity (10). Therefore, we tested fluorescent bead phagocytosis following exposure to combinations of IFNβ, TNFSF15, TNFα, and IL1β. To improve statistical power and in view of the lack of effect on maturation markers, IL28 was excluded from this study. Cells were pretreated with cytokine combinations for 8 h, and then co-cultured with fluorescent beads for 4 h. The number of beads in each cell was then counted using imaging flow cytometry. The highest rates of phagocytosis were seen with all four cytokines exposed together and with exposure to IFNβ with either IL1β or TNFSF15 (Figure 6E).

**Figure 7:**
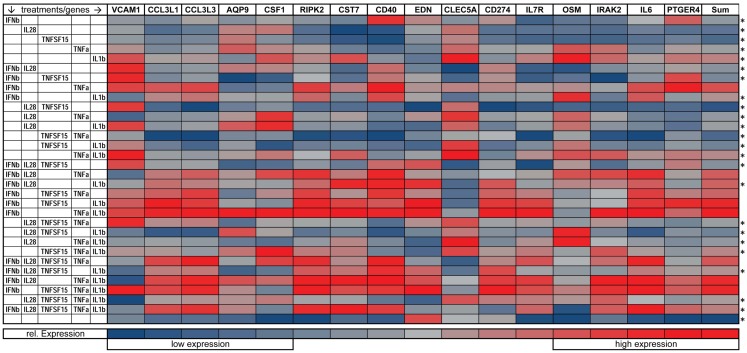
**Gene expression of immune relevant genes after combinatorial IFNβ, IL28, TNFSF15, TNFα, and IL1β treatment**. Heat map is normalized to the lowest (blue) and highest (red) expression level for each individual gene (*p ≤ 0.05 compared to cells exposed to the triple combination of TNFα, IFNβ, and IL1b).

We also looked at the capacity of combinations of the five cytokines to regulate 16 immune related genes that had been identified as preferentially induced by paracrine signaling in comparison with IFNβ exposure alone (see Figure 1C). Here, we exposed DCs for 8 h to all possible combinations of IFNβ, IL28, TNFSF15, TNFα, IL1β, mRNA, and performed qPCR. Gene expression is plotted on a heatmap (Figure 7). The genes assayed were *VCAM-1*, *AQP9*, *RIPK2*, *IRAK2*, *CCL3L1*, *IL6*, *OSM*, *CCL3L3*, *CSF1*, *CD274*, *CD40*, *IL7R*, *CLEC5A*, *EDN*, *CST7*, and *PTGER4*). Nine of these genes showed the highest induction when treated with the triple combination of IFNβ, TNFα, and IL1β (Figure 7). The genes showing combinatorial cytokine preferences are associated with anti-viral immunity (*CCL3L3*, *CSF1*, *IL7R*), regulation of inflammation (*AQP9*, *IRAK2*, *RIPK2*), and immune cell activation (*CST7*, *EDN*, cytokine *OSM*). The induction of IL6 was also high with the triple cytokine treatment. Overall, these experiments demonstrate that IFNβ, TNFα, and IL1β acting together are the principal drivers of the paracrine induced anti-viral state in DCs.

**Figure 8:**
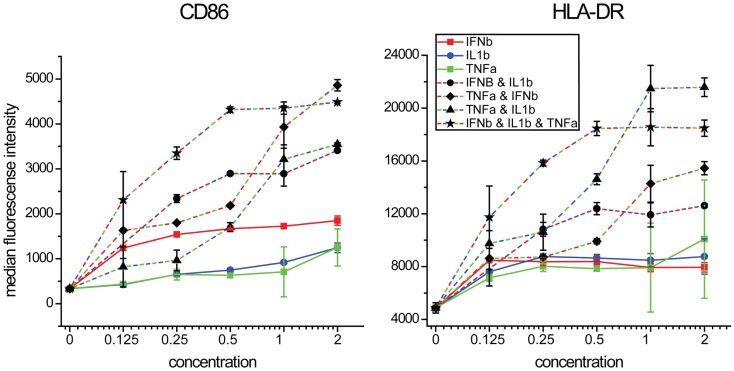
**Maturation marker expression by combinations of IFNβ, TNFα, and IL1β**. DCs were exposed to combinations of three cytokines at a range of concentrations relative to that found in supernatants of NDV infected DCs.

We next performed concentration response studies to determine if the combinatorial effects of the cytokines were synergistic. At lower concentrations, all three cytokines together produced the highest levels of both CD86 expression although at higher concentrations, equivalent levels could be achieved with the combination of TNFα and IFNβ alone (Figure 8). For HLA-DR expression, the combination of all three cytokines at lower concentrations produced the highest levels, although at higher concentrations TNFα and IL1β induced similar levels. For both maturation markers, the effects of combinatorial exposure dramatically exceeded the effects of any individual cytokine. Statistical values for pairwise comparisons within each dilution step are shown in Tables S1 and S2 in Supplementary Material. We also investigated the effect of combinations of various combinations of the two cytokines IFNβ and TNFα, which showed a synergistic induction with CD86 (Figure S3 in Supplementary Material). These results support the view that the effects of combinatorial exposure produce a qualitatively different cellular effect than do the individual cytokines.

### Combinatorial effects of TNFα, IL1β, and IFNβ on pathogenic viruses

**Figure 9:**
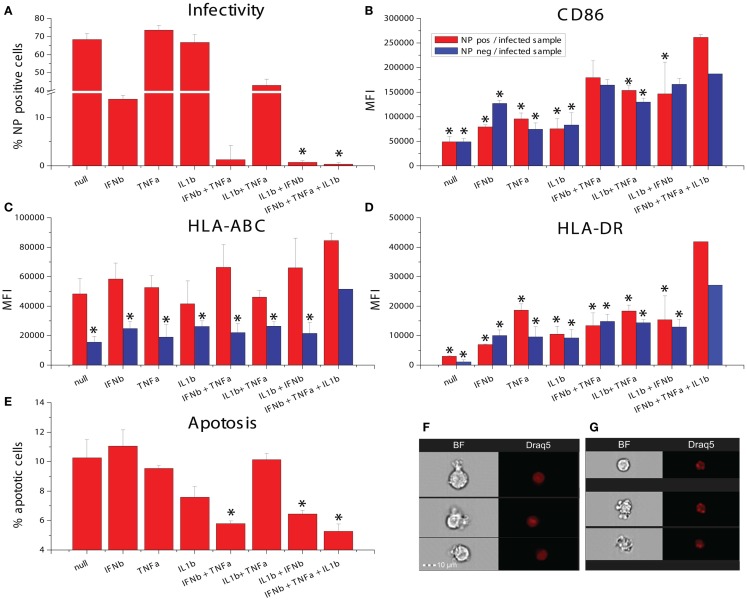
**Effects of combinatorial of IFNβ, TNFα, and IL1β treatment on DCs infected with the pandemic influenza A Cal/09 strain**. **(A)** Infectivity assayed by NP expression (*p ≤ 0.05 compared to cells exposed to IFNβ). **(B)** Expression of the maturation marker CD86. **(C)** Expression of the maturation marker HLA-ABC. **(D)** Expression of the maturation marker HLA-DR (*p ≤ 0.05 compared to cells exposed to the triple combination of TNFα, IFNβ, and IL1b). **(E)** Apoptosis assayed by assessing nuclear fragmentation and cell granularity by imaging flow cytometry (*p ≤ 0.05 compared to untreated cells). **(F)** Sample images of non-apoptotic cells. **(G)** Sample images of apoptotic cells.

The studies described above rely on NDV, which is not pathogenic in humans and does not express immune antagonists having activity in human cells. We were interested in studying the effects of combination cytokine exposure on a human pathogen and studied the recent pandemic Influenza A virus Cal/09. We pretreated DCs with all combinations of IFNβ, TNFα, and IL1β for 8 h and subsequently infected them with influenza A/California/7/2009 and measured infectivity, maturation marker induction as well as induction of apoptosis. The triple combination of all three cytokines as well as the dual cytokine mixture of IL1β and IFNβ significantly increased the suppression of infectivity when compared to IFNβ pretreatment alone (Figure 9A). The triple combination also caused the highest induction of CD86, HLA-ABC, and HLA-DR in virus NP-expressing cells (Figures 9B–D). Apoptosis was assayed in the same samples using imaging flow cytometry to measure nuclear fragmentation (Figures 9F,G). Interestingly, pretreatment with single cytokines did not reduce influenza-induced apoptosis, whereas the triple combination as well as the dual cytokine combinations with IFNβ could reduce cell death (Figure 9E). These data suggests that the triple combination of cytokines improves DC survival, resistance to infectivity, and increases costimulatory marker expression for T-cell activation.

### Combinatorial effect of TNFα, IL1β, and IFNβ on induction of virus specific T-cell response

**Figure 10:**
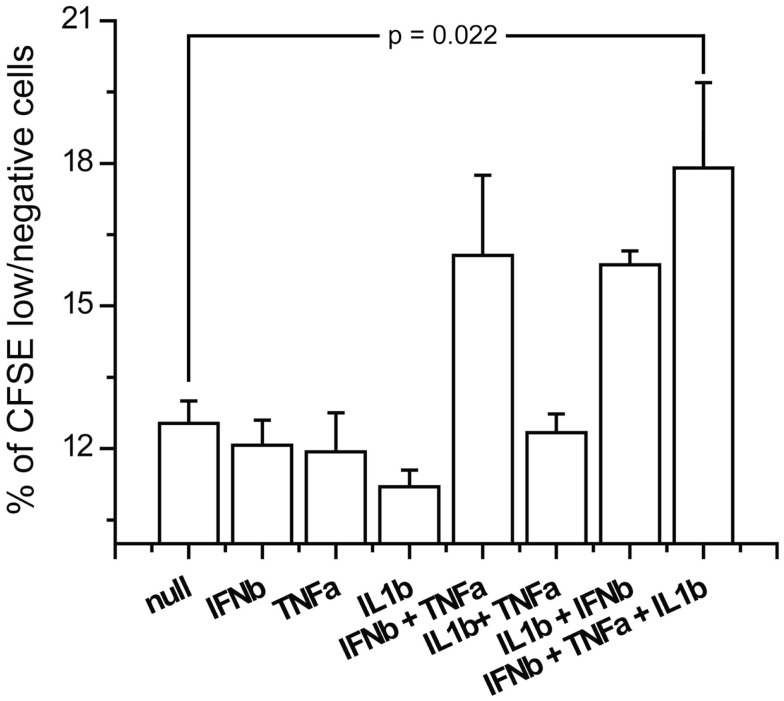
**Antigen specific proliferation of T-cells exposed to cytokine pretreated DCs**. Measles antilogous specific T-cells were co-cultured with cytokine pretreated DCs which were also pulsed with measles vaccine. Proliferation was measured by the dilution of CFSE.

In order to see if the induction of the costimulatory markers by the triple combination of IFNβ, TNFα, and IL1β has an effect on T-cell activation, we studied the induction of measles specific T-cell proliferation after co-culture with cytokine pretreated and measles primed T-cells. We exposed the CD14 depleted PBMCs from the same donors which were used for DC generation to measles vaccine and harvested T-cell 5 days later. Those T-cells were co-cultured for 3 days with measles primed DCs exposed to combinations of IFNβ, TNFα and IL1β. Proliferation was measured by the dilution of the membrane bound dye CFSE by flow cytometry. The triple combination of IFNβ, TNFα, and IL1β significantly increased cell proliferation compared to non-pretreated DCs (Figure 10). These results indicate that the costimulatory marker upregulation also affects activation of the adaptive immune system.

### Combinatorial effect of TNFα, IL1β, and IFNβ on influenza morbidity *in vivo*

**Figure 11:**
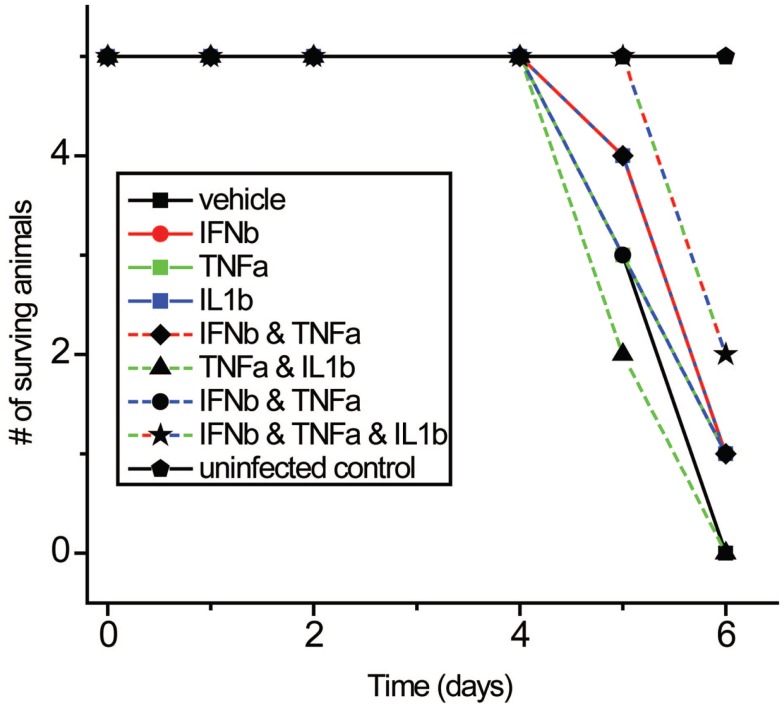
**Effects of IFNβ, TNFα, and IL1β combinations on mortality with PR8 virus *in vivo***. Mice received cytokine injection i.p. 6 h before and 6 h after infection with PR8 virus.

We next studied the effects of IFNβ, TNFα, and IL1β on influenza virus pathogenicity *in vivo* using a well-characterized aerosolized-virus mouse infection model (69). Cytokines were injected intraperitoneally both 3 h before and after inhalation infection with PR8 virus. While the differences were modest, the triple combination was found to improve survival times significantly compared to control (*p*: 0.0456) following PR8 infection in mice (Figure 11).

## Discussion

In this study, we show that IFNβ, TNFα, and IL1β are secreted by virus-infected DCs and act combinatorially to alter the anti-viral response state of uninfected DCs. This combination is responsible for maturation marker upregulation in naive as well as in infected cells, reduction of virus induced apoptosis, heightened phagocytic activity, specific autologous T-cell activation, and resistance to viral infection *in vitro* as well as in *in vivo*.

The importance of cellular micro-environments in dictating immune cell responses is supported by the report that the inflammatory state of macrophages can be reprogramed by exposure to an anti- or pro-inflammatory stimuli (70). Another report has suggested that the initial exposure to a cytokine signal determines and fixes the final state of the macrophage (71). These reports, as well as the finding that the combination of IL-4, IL-10, and TGFb skew the development of myeloid cells into M2 macrophages, support the importance of combinatorial cytokine signals in immune regulation (72). DCs themselves are differentiated into different lineages by exposure to different cytokines including GMCSF and Flt3 (73).

Since the discovery of type I interferon, paracrine cytokine signaling has been recognized as a crucial component in orchestrating the immune responses to virus infection. Recent studies have begun to reveal the importance of combinatorial extracellular stimuli in directing the responses of immune cells. For example, when DCs are exposed to lipopolysaccharide in the context of apoptotic cells, they induce TH17 cells, a response that is not achieved by either stimulus alone (74). Retinoic acid acts alone on T-cells to induce Treg cells. However, retinoic acid combined with IL-15 causes DCs to skew the T-cell polarization toward TH17 cells (6). TLR7/8 ligand combined with either TLR3 or TLR4 ligands synergistically increases IFNβ and IFNλ1 expression in DCs (75). SCF and IL-2 have a synergistic effect on the proliferation NK cells (7). TNFα and IFNγ act together on smooth airway cells to enhance CXCL-10 expression (8). IL17 together with TNFα or IL1β induces MCP-1 and MIP-2 in murine mesangial cells (9). These combinatorial effects are likely to prove clinically relevant, for example, by contributing to individual differences in the response to cytokine treatment (76). While beyond the scope of the present investigation, the role of relative timing of combinatorial cytokine signals is another important area for further study. We have also not addressed the potential combinatorial role of alarmins, which can work in concert with cytokines to induce different cell states (77).

To our knowledge, this is the first report of IFNβ, TNFα, and IL1β working in concert to alter the response state of any immune cell. Previous studies have implicated pairs of this triad in influencing immune responses. The combination of IL-1β and IFNβ has been reported to promote immune control of West Nile virus infection in the CNS (78). TNFα and IFNβ have also been found to affect macrophages and fibroblasts in reducing the infectivity of poxviruses (79, 80).

Several of the transcripts that are preferentially induced in DCs by the exposure to all three cytokines have been found to serve important roles in inflammation and immunity: CCL3L3 suppresses HIV proliferation (23); AQP9 is a marker for inflammation (81); CSF1 is a biomarker for respiratory syncytial virus infection (24); RIPK2 knockout in mice causes hyper-susceptibility to infection with influenza A virus (82); EDN possesses anti-viral activity against single stranded RNA viruses like respiratory syncytial virus, Hepatitis and HIV (83); IL7R expression inversely correlates with FoxP3 and suppressive function of human CD4^+^ T reg cells (84); OSM is a pro-inflammatory cytokine (22); and IRAK2 is needed to sustain cytokine production during prolonged activation of the TLR signaling pathway (85). When maturation marker induction was studied by cytokine induction alone, we found that CD86 is most strongly driven by IFNβ with synergistic effects of TNFα or IL1β. HLA-DR was little changed by individual cytokines but was strongly induced by TNFα and IL1β together. The gene VCAM-1 was most induced by the TNFα and IL1β together and PTGER4 was most induced by IFNβ and TNFα. While the overall DC cell state observed requires all three cytokines, the differences in the cytokines most important for various components of these DC responses provides the basis for future studies to dissect the underlying signaling and transcriptional mechanisms involved in these combinatorial effects.

Surprisingly, the triple combination of IFNβ, TNFα, and IL1β reduced influenza-induced cell death in infected DCs. This is interesting as IFNβ is known to be an inducer of apoptosis in DCs (86), and indicates how the effects of one cytokine may be very different depending on which other cytokines are stimulating a cell. Maturation marker induction as well as cell survival are important for the activation of the adaptive immune system. The observation of a heightened proliferation of virus specific T-cells when exposed to DCs pretreated with the triple combination supports this view. The modestly increased survival of mice to PR8 infection when treated with the three cytokines suggests the combinatorial coding of cell responses has significance *in vivo*.

The large number of cytokines secreted by infected DCs is remarkable. We identify combinatorial effects involving only three of these secreted factors on DCs. It is probable that combinatorial signaling of different cytokine mixtures influences the activation state of other immune cells and that other immune cells also serve as the source of complex cytokine signals. The specific activation state of any immune cell can depend on both the cytokine mixture present and their concentration (see Figure 8). Thus the immune system can potentially generate many distinct micro-environments that shape the local activation state of various immune cells. This provides the potential for a dynamic and spatially distributed complexity of the set point of the immune system that could be crucial in organizing the local and system responses to infection, neoplasia, and injury. Unraveling this combinatorial code may have benefits in guiding combination immunotherapy for autoimmune diseases, for chronic infections and for other immune system influenced diseases as well as for personalizing interventions in relationship to individual variation in background cytokine expression.

## Conflict of Interest Statement

The authors declare that the research was conducted in the absence of any commercial or financial relationships that could be construed as a potential conflict of interest.

## Supplementary Material

The Supplementary Material for this article can be found online at http://www.frontiersin.org/Journal/10.3389/fimmu.2014.00073/abstract
