# Supplementary material for: Combinatorial Cytokine Code Generates Anti-Viral State in Dendritic Cells
Source: Front Immunol. 2014 Feb 26;5:73. doi: 10.3389/fimmu.2014.00073 (PMC3935347; doi:10.3389/fimmu.2014.00073)
Supplement: Supplementary file 1 [file 71252_Sealfon_DataSheet1.PDF]

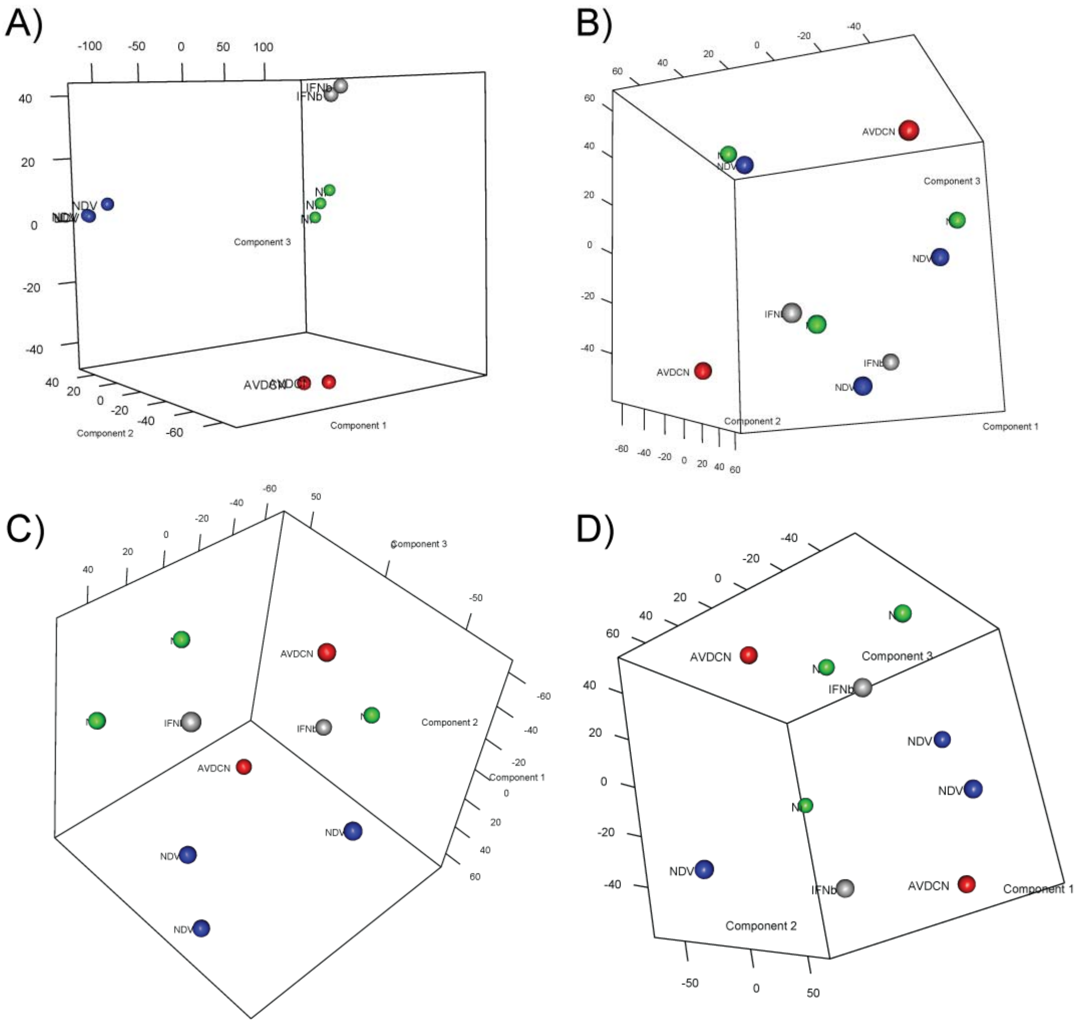

Figure S1: Robustness analysis of PCA. Randomized design is generally considered as one the most robust evaluation methodologies. We used it here to evaluate the robustness of the PCA analysis. An expression vector for each probe ID was randomly shuffled and the PCA analysis was repeated on the randomize data. The scores of the first three components of the PCA based on the original are depicted in Fig S1A and and on the randomized data S1B, S1C and S1B correspondingly.

To test for robustness we used the statistical MANOVA analysis to test for presence of clusters both in our and in the randomized data set. Our null hypothesis was that the data doesn't contain any clusters. Formally, we tested to see if the vector of values of the dependent variables (coordinates) is equal for multiple independent groups (samples) and our null hypothesis would be:

$$H0 : \begin{pmatrix} X11 \\ Xp1 \end{pmatrix} = \begin{pmatrix} X12 \\ Xp2 \end{pmatrix} = \ldots = \begin{pmatrix} X1k \\ Xpk \end{pmatrix},$$

where p represents the total number of dependent variables (coordinates) for k levels (samples). The Wilks test for indicated that the null hypothesis can be rejected with p-value of 5.08e-15, indicating the presence of clusters in the data. The same test on the randomized data fails to rejects the null hypothesis (p-value of 0.472), indicating that the randomized data set doesn't include clusters.

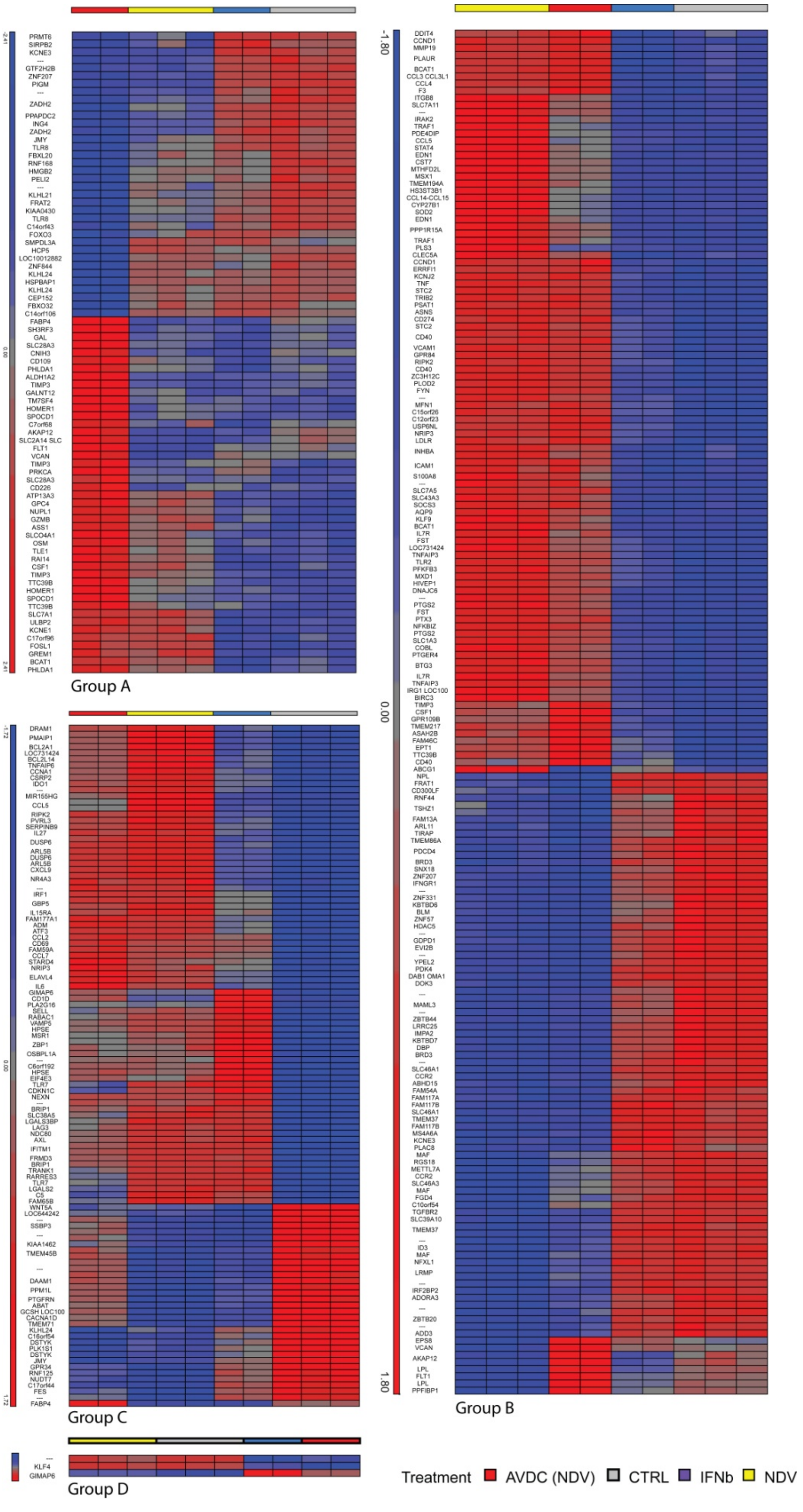

Figure S2: Transcripts which showed a significant change between exposure to paracrine signaling and single cytokine IFNb treatment. Group (A): Transcripts significantly altered by the paracrine signaling that did not show significant induction by NDV infection or IFNb treatment when compared to control. Group (B): Transcripts significantly induced by paracrine signaling and NDV infection but not IFNb treatment when compared to control. Group (C): Transcripts significantly induced by paracrine signaling, NDV infection and IFNb treatment when compared to control. Group (D): Transcripts significantly altered by paracrine signaling and IFNb treatment when compared to control that also differed significantly between exposure to paracrine signaling and IFNb treatment.

Table S1: Pair wise comparisons for CD86 of DCs treated in all combinations with IFNβ, TNFα and IL1b. Pair wise comparisons were calculated with ANOVA followed by Tukey's 'Honest Significant Difference' method.

	CD86 1:1				CD86 1:2				CD86 1:4				CD86 1:8			
	diff	lwr	upr	p adj	diff	lwr	upr	p adj	diff	lwr	upr	p adj	diff	lwr	upr	p adj
IFNb/IL1b-IFNb	1523	837.02	2208	9.57E-06	1222	-84.49	2528	0.076964	1181	945.4	1417	1.51E-11	732.88	233.68	1232.1	0.001743
IL1b-IFNb	-644	-1329	41.82	0.075132	-771	-2078	534.7	0.505333	-833	-1069	-597	6.65E-09	-842.91	-1342.1	-343.71	0.000356
null-IFNb	-1418	-2012	-825	3.32E-06	-1296	-2427	-165	0.018106	-1229	-1433	-1025	4.85E-13	-1122.6	-1554.9	-690.29	9.46E-07
TNFa-IFNb	-215	-900.6	470.5	0.957043	-463	-1769	843.6	0.921991	-975	-1210	-739	4.60E-10	-761.11	-1260.3	-261.92	0.001155
TNFa/IFNb-IFNb	2946	2260.6	3632	2.34E-10	2128	821.83	3434	0.000544	454.4	218.5	690.2	6.76E-05	221.26	-277.94	720.45	0.797043
TNFa/IL1b-IFNb	1721	1035.1	2406	1.59E-06	1303	-2.767	2610	0.050723	-82.5	-318	153.4	0.926641	-386.8	-885.99	112.4	0.206002
TNFa/IL1b/IFNb-IFNb	2642	1956.1	3327	1.51E-09	2741	1435.2	4048	2.12E-05	2665	2429	2901	0	1867	1367.8	2366.2	2.52E-09
IL1b-IFNb/IL1b	-2166	-2852	-1481	4.21E-08	-1993	-3299	-687	0.001145	-2014	-2250	-1778	0	-1575.8	-2075	-1076.6	4.29E-08
null-IFNb/IL1b	-2941	-3534	-2347	1.86E-11	-2518	-3649	-1386	9.29E-06	-2410	-2614	-2206	0	-1855.5	-2287.8	-1423.2	2.39E-10
TNFa-IFNb/IL1b	-1738	-2423	-1052	1.37E-06	-1684	-2990	-378	0.006411	-2156	-2392	-1920	0	-1494	-1993.2	-994.79	1.02E-07
TNFa/IFNb-IFNb/IL1b	1424	738.11	2109	2.45E-05	906.3	-399.8	2212	0.317382	-727	-963	-491	6.33E-08	-511.62	-1010.8	-12.427	0.042191
TNFa/IL1b-IFNb/IL1b	198.1	-487.4	883.6	0.972061	81.72	-1224	1388	0.999999	-1264	-1500	-1028	4.16E-12	-1119.7	-1618.9	-620.48	8.31E-06
TNFa/IL1b/IFNb-IFNb/IL1b	1119	433.6	1805	0.000531	1520	213.57	2826	0.015933	1484	1248	1720	2.23E-13	1134.1	634.89	1633.3	6.92E-06
null-IL1b	-775	-1368	-181	0.005743	-524	-1656	606.8	0.760523	-396	-600	-192	6.24E-05	-279.7	-712.02	152.61	0.397257
TNFa-IL1b	428.7	-256.9	1114	0.437538	308.9	-997.3	1615	0.990936	-142	-377	94.28	0.486079	81.797	-417.4	580.99	0.999054
TNFa/IFNb-IL1b	3590	2904.3	4275	6.46E-12	2899	1593.3	4206	9.64E-06	1287	1051	1523	2.92E-12	1064.2	564.97	1563.4	1.71E-05
TNFa/IL1b-IL1b	2364	1678.8	3050	9.89E-09	2075	768.72	3381	0.000728	750.5	514.6	986.4	3.77E-08	456.11	-43.083	955.3	0.08857
TNFa/IL1b/IFNb-IL1b	3285	2599.8	3971	3.42E-11	3513	2206.7	4819	5.51E-07	3498	3262	3734	0	2709.9	2210.7	3209.1	3.23E-12
TNFa-null	1203	609.48	1797	3.43E-05	833.3	-297.9	1964	0.254232	254.3	50.01	458.6	0.008869	361.5	-70.816	793.81	0.143841
TNFa/IFNb-null	4364	3770.7	4958	0	3424	2292.7	4555	8.48E-08	1683	1479	1887	0	1343.9	911.55	1776.2	5.51E-08
TNFa/IL1b-null	3139	2545.1	3732	5.38E-12	2599	1468.1	3730	5.86E-06	1146	942.1	1351	1.72E-12	735.81	303.5	1168.1	0.000323
TNFa/IL1b/IFNb-null	4060	3466.2	4654	4.31E-14	4037	2906.1	5168	5.56E-09	3894	3690	4098	0	2989.6	2557.3	3421.9	3.20E-14
TNFa/IFNb-TNFa	3161	2475.7	3847	6.84E-11	2591	1284.4	3897	4.59E-05	1429	1193	1665	4.29E-13	982.37	483.17	1481.6	5.09E-05
TNFa/IL1b-TNFa	1936	1250.1	2621	2.57E-07	1766	459.83	3072	0.004063	892.1	656.2	1128	2.08E-09	374.31	-124.88	873.51	0.236722
TNFa/IL1b/IFNb-TNFa	2857	2171.2	3542	3.97E-10	3204	1897.8	4510	2.24E-06	3640	3404	3875	0	2628.1	2128.9	3127.3	5.84E-12
TNFa/IL1b-TNFa/IFNb	-1226	-1911	-540	0.000176	-825	-2131	481.6	0.426185	-537	-773	-301	6.74E-06	-608.05	-1107.2	-108.86	0.010761
TNFa/IL1b/IFNb-TNFa/IFNb	-305	-990	381	0.795358	613.4	-692.8	1920	0.749257	2211	1975	2447	0	1645.7	1146.5	2144.9	2.10E-08
TNFa/IL1b/IFNb-TNFa/IL1b	921	235.51	1607	0.004323	1438	131.84	2744	0.024853	2747	2512	2983	0	2253.8	1754.6	2752.9	9.94E-11

Table S2: Pair wise comparisons for CD86 of DCs treated in all combinations with IFNβ, TNFα and IL1b. Pair wise comparisons were calculated with ANOVA followed by Tukey's 'Honest Significant Difference' method.

	HLA-DR 1:1				HLA-DR 1:2				HLA-DR 1:4				HLA-DR 1:8			
Comparisons	diff	lwr	upr	p adj	diff	lwr	upr	p adj	diff	lwr	upr	p adj	diff	lwr	upr	p adj
IFNb/IL1b-IFNb	4589	-2547	11725	0.40441	4279	-2652	11209	0.452785	4255	2500	6011	2.67E-06	2574.6	29.2	5120.1	0.046248
IL1b-IFNb	1478	-5659	8614	0.995899	865	-6065	7796	0.999841	707	-1049	2462	0.861868	602.3	-1943.1	3147.8	0.990902
null-IFNb	-2900	-9080	3280	0.749974	-2968	-8970	3034	0.701285	-3058	-4578	-1537	3.80E-05	-2936.0	-5140.4	-731.6	0.004708
TNFa-IFNb	5854	-1283	12990	0.158249	2887	-4043	9818	0.840835	-474	-2230	1281	0.980642	309.4	-2236.0	2854.8	0.999866
TNFa/IFNb-IFNb	7076	-60	14213	0.052935	5927	-1004	12858	0.128117	1392	-363	3148	0.185472	523.8	-2021.7	3069.2	0.996056
TNFa/IL1b-IFNb	13705	6568	20841	7.04E-05	12030	5099	18960	0.000254	6190	4435	7946	6.82E-09	3509.1	963.6	6054.5	0.003348
TNFa/IL1b/IFNb-IFNb	10281	3145	17418	0.002128	11647	4716	18577	0.000377	10675	8920	12430	4.02E-13	7832.5	5287.1	10378.0	6.50E-08
IL1b-IFNb/IL1b	-3111	-10248	4025	0.809459	-3413	-10344	3517	0.705152	-3549	-5304	-1793	3.55E-05	-1972.3	-4517.7	573.1	0.20601
null-IFNb/IL1b	-7489	-13669	-1309	0.011261	-7246	-13248	-1244	0.011632	-7313	-8833	-5793	3.20E-11	-5510.6	-7715.0	-3306.2	1.69E-06
TNFa-IFNb/IL1b	1265	-5872	8401	0.998442	-1391	-8322	5540	0.996612	-4730	-6485	-2974	5.36E-07	-2265.2	-4810.7	280.2	0.103112
TNFa/IFNb-IFNb/IL1b	2487	-4649	9623	0.927716	1648	-5282	8579	0.99063	-2863	-4618	-1108	0.000537	-2050.9	-4596.3	494.6	0.172477
TNFa/IL1b-IFNb/IL1b	9116	1979	16252	0.007003	7751	820	14682	0.021971	1935	179	3690	0.024654	934.4	-1611.0	3479.9	0.907876
TNFa/IL1b/IFNb-IFNb/IL1b	5692	-1444	12828	0.180809	7368	438	14299	0.032391	6420	4664	8175	3.68E-09	5257.9	2712.4	7803.3	2.64E-05
null-IL1b	-4378	-10558	1802	0.294912	-3833	-9835	2169	0.412601	-3764	-5285	-2244	1.95E-06	-3538.3	-5742.7	-1333.9	0.000646
TNFa-IL1b	4376	-2760	11512	0.460796	2022	-4908	8953	0.970581	-1181	-2936	575	0.352093	-292.9	-2838.4	2252.5	0.999907
TNFa/IFNb-IL1b	5598	-1538	12735	0.195019	5062	-1869	11992	0.262963	686	-1070	2441	0.878488	-78.6	-2624.0	2466.9	1
TNFa/IL1b-IL1b	12227	5090	19363	0.000298	11165	4234	18095	0.00062	5483	3728	7239	5.09E-08	2906.7	361.3	5452.2	0.018587
TNFa/IL1b/IFNb-IL1b	8803	1667	15940	0.009619	10782	3851	17712	0.000923	9968	8213	11724	1.38E-12	7230.2	4684.7	9775.6	2.34E-07
TNFa-null	8754	2574	14934	0.00254	5855	-147	11857	0.058972	2583	1063	4104	0.00033	3245.4	1041.0	5449.8	0.001691
TNFa/IFNb-null	9976	3796	16157	0.000605	8895	2893	14897	0.001576	4450	2930	5970	1.45E-07	3459.8	1255.3	5664.2	0.000835
TNFa/IL1b-null	16605	10424	22785	5.60E-07	14997	8995	21000	1.70E-06	9248	7727	10768	3.99E-13	6445.0	4240.6	8649.5	1.48E-07
TNFa/IL1b/IFNb-null	13181	7001	19361	1.70E-05	14615	8612	20617	2.50E-06	13733	12212	15253	0.00	10768.5	8564.1	12972.9	2.41E-11
TNFa/IFNb-TNFa	1222	-5914	8359	0.998744	3039	-3891	9970	0.80508	1867	111	3622	0.032335	214.4	-2331.1	2759.8	0.999989
TNFa/IL1b-TNFa	7851	714	14987	0.024997	9142	2212	16073	0.005167	6664	4909	8420	1.95E-09	3199.6	654.2	5745.1	0.008117
TNFa/IL1b/IFNb-TNFa	4427	-2709	11564	0.446906	8759	1829	15690	0.007724	11149	9394	12905	1.89E-13	7523.1	4977.7	10068.5	1.24E-07
TNFa/IL1b-TNFa/IFNb	6628	-508	13765	0.080292	6103	-828	13034	0.109391	4798	3042	6553	4.29E-07	2985.3	439.8	5530.7	0.014907
TNFa/IL1b/IFNb-TNFa/IFNb	3205	-3931	10341	0.786751	5720	-1211	12651	0.15358	9283	7527	11038	5.36E-12	7308.7	4763.3	9854.2	1.97E-07
TNFa/IL1b/IFNb-TNFa/IL1b	-3424	-10560	3713	0.730042	-383	-7314	6548	0.999999	4485	2729	6240	1.21E-06	4323.5	1778.0	6868.9	0.000331

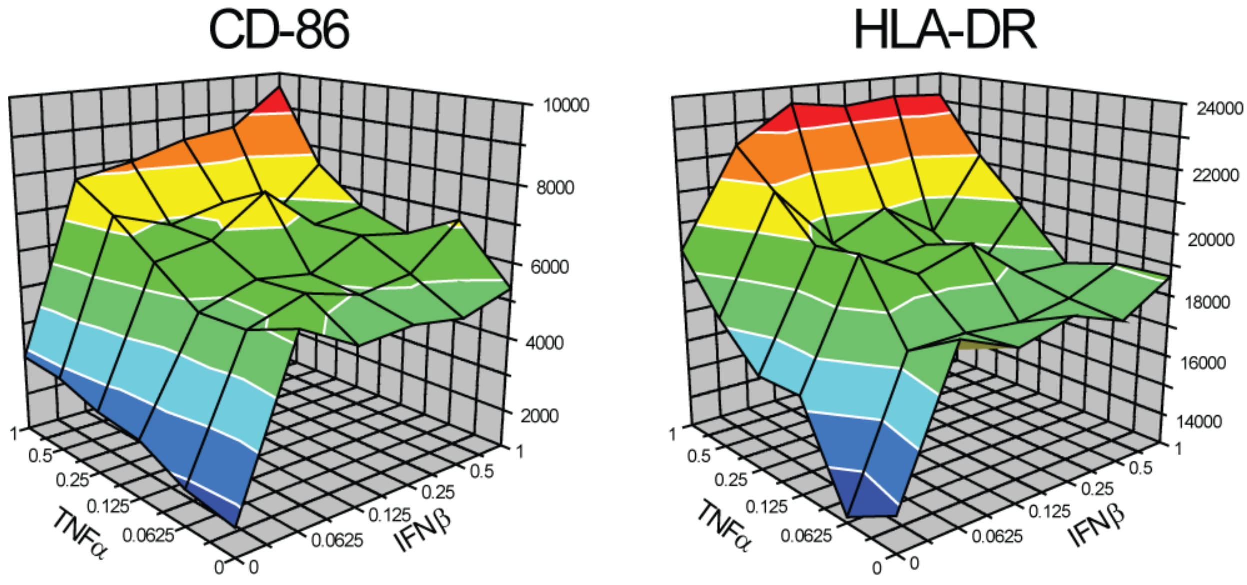

Figure S3: Combinatorial effects of IFNβ and TNFα on DC maturation marker expression. DCs were exposed to all possible combinations of dilutions of IFNβ and TNFα for 8 hours. Maturation marker expression was measured by flow cytometry

Table S3: Table with primers used in analysis

GENE	Sense	Antisense
CXCL1	CACTGCGCCCAAACCGAAGT	TCCCTTCTGGTCAGTTGGAT
IL28	GCCTTTAAGAGGGCCAAAGA	GAGGATATGGTGCAGGGTGT
TNFSF4	CATGGTCCCCTCTCTTAGGT	TGGAGTGAGGCTGGTGCATA
TNFSF15	GGGTTGGAGGATTGGCGAGT	CTGGACCACTGGTGACCATT
IL29	GCCTCCTCACGCGAGACCTC	GGAGTAGGGCTCAGCGCATA
IL18	GAGGATGATGAATTGGGGGATAG	TAGCTGGGATTGAGGGCATG
IFNa16	GGTGTTTGATGGCAACCAGT	GTCACACAGGCTTCTAGGTC
VCAM1	TCAGATTGGTGACTCCGTCT	TCACCTTCCCATTCAGTGGA
CCL3L1	GTGCTCCAAGCCCAGTGTCA	TCTGGACCCCTCAGGCACTC
CCL3L3	GTGCTCCAAGCCCAGTGTCA	AGGTCACACGCATGTTCCCA
AQP9	AGAGAAGCCCCAAGATGCAG	CACAGCCACATCCAAGGACA
CSF1	ACCCGCAGACCCTTGACTGA	TCACCAGCCTTCCTCAAGCA
RIPK2	CCAAGCCTACAAGGACCTCA	GGTAAGGCTGAAGACCCATT
CST7	TGTTCAAGGAGTCCCGCATC	ACAGTCATCCAGACGCAGGT
CD40	AGAACCTCTCACTTCACCCT	ACCAAACTGCTGGATCGGAA
EDN	CAGCAGAAACACACAGTCACA	ACACTCCCTTAGGACCTTCGT
CLECA5A	GAGATCAGCAGAGCCACCCTA	GCCAGATGGAGATTGCATCAGT
CD274	GCCAGAAAAGCCTCATTCGT	CCCAGGGCATCTGAATCTCG
IL7R	GGACCTCCTGCTTAGCCTT	TCATCTTTGTCGCTCACGGTA
OSM	GCCTCATCATGTCCCAAACC	ATCCAAGCAACCGACAGGCA
IRAK2	AGTCACTGCCTGGTACCTT	CAGGTGCCTACAGTCCCAT
IL6	AATGCCAGCCTGCTGACGAA	CTGAGGTGCCCATGCTACAT
PTGER4	ATTCGTCCGCCTCCTTGAGC	ACAGCCAGCCCACATACCAG
